# Metformin inhibits cell cycle progression of B-cell chronic lymphocytic leukemia cells

**DOI:** 10.18632/oncotarget.4168

**Published:** 2015-06-05

**Authors:** Silvia Bruno, Bernardetta Ledda, Claudya Tenca, Silvia Ravera, Anna Maria Orengo, Andrea Nicola Mazzarello, Elisa Pesenti, Salvatore Casciaro, Omar Racchi, Fabio Ghiotto, Cecilia Marini, Gianmario Sambuceti, Andrea DeCensi, Franco Fais

**Affiliations:** ^1^ Department of Experimental Medicine, University of Genoa, Genoa, Italy; ^2^ Department of Pharmacology, University of Genova, Genova, Italy; ^3^ IRCCS AOU San Martino - IST Istituto Nazionale per la Ricerca sul Cancro, Genova, Italy; ^4^ The Feinstein Institute for Medical Research, North Shore-Long Island, Experimental Immunology, Manhasset, NY, USA; ^5^ Department of Internal Medicine and Medical Specialty, University of Genova, Genova, Italy; ^6^ Hematology-Oncology Unit - Ospedale Villa Scassi, Genova, Italy; ^7^ CNR Institute of Bioimages and Molecular Physiology, Milan, Section of Genoa, Genoa, Italy; ^8^ Department of Health Science, University of Genova, Genova, Italy; ^9^ Division of Cancer Prevention and Genetics, European Institute of Oncology, Milan, Italy; ^10^ Division of Medical Oncology, Ospedali Galliera, Genova, Italy

**Keywords:** metformin, cell proliferation, cell activation, chronic lymphocytic leukemia, cancer therapy

## Abstract

B-cell chronic lymphocytic leukemia (CLL) was believed to result from clonal accumulation of resting apoptosis-resistant malignant B lymphocytes. However, it became increasingly clear that CLL cells undergo, during their life, iterative cycles of re-activation and subsequent clonal expansion. Drugs interfering with CLL cell cycle entry would be greatly beneficial in the treatment of this disease.

1, 1-Dimethylbiguanide hydrochloride (metformin), the most widely prescribed oral hypoglycemic agent, inexpensive and well tolerated, has recently received increased attention for its potential antitumor activity. We wondered whether metformin has apoptotic and anti-proliferative activity on leukemic cells derived from CLL patients.

Metformin was administered *in vitro* either to quiescent cells or during CLL cell activation stimuli, provided by classical co-culturing with CD40L-expressing fibroblasts.

At doses that were totally ineffective on normal lymphocytes, metformin induced apoptosis of quiescent CLL cells and inhibition of cell cycle entry when CLL were stimulated by CD40-CD40L ligation. This cytostatic effect was accompanied by decreased expression of survival- and proliferation-associated proteins, inhibition of signaling pathways involved in CLL disease progression and decreased intracellular glucose available for glycolysis.

In drug combination experiments, metformin lowered the apoptotic threshold and potentiated the cytotoxic effects of classical and novel antitumor molecules.

Our results indicate that, while CLL cells after stimulation are in the process of building their full survival and cycling armamentarium, the presence of metformin affects this process.

## INTRODUCTION

B-cell chronic lymphocytic leukemia (CLL) is the most common leukemia in human adults of the Western world and no definitive cure is yet available.

CLL was believed for long time to be the result of the clonal accumulation of resting and apoptosis-resistant malignant B lymphocytes. Indeed, the majority of peripheral blood CLL cells are arrested in G0/G1 cell cycle phase and show a gene expression profile of resting cells [1]. However, studies on the biology of the disease undertaken in the last decade, have provided a new, more dynamic, view. *In vivo* deuterium (2H) labeling of CLL cells [2, 3] revealed substantial birth rates of CLL cells, which vary from 0.08% to 1.7% of the entire clone per day, with higher birth rates associating with more aggressive disease [2]. Studies on samples obtained from blood, bone marrow, and lymph nodes showed that proliferating leukemic cells were indeed present, particularly in lymph nodes [2, 4, 5], supporting the notion that activation and clonal expansion take place in lymphoid proliferation centers mostly within secondary lymphoid tissues, where multiple molecular interactions with antigen and microenvironment contribute to leukemic B cell survival and proliferation. Yet, the peripheral blood contains intraclonal dynamic subpopulations of leukemic cells with different molecular characteristics marking the timing of previous activation [6–8]. Analysis of these subpopulations revealed a spectrum of leukemic cells ranging from recently divided cells that are lymphoid tissue emigrants, to ‘older’ cells that will either reentry into lymphoid tissue or die [7, 8]. Importantly, when transferred *in vitro* and stimulated by microenvironment-simulating signals the leukemic cells from the peripheral blood retain the capability of reentering the cell cycle [9, 10]. Taken together, these results indicate a dynamic picture where CLL cells traffic between peripheral blood and lymphoid tissues, undergo iterative rounds of slowing-down to quiescence in the periphery and re-activation with subsequent clonal expansion in lymphoid tissues. Changes of cytogenetic abnormalities and acquisition of new chromosomal defects observed during progression of CLL [11, 12] further endorse the notion that cyclic (multiple?) rounds of leukemic cell stimulation occur during and concur to disease evolution.

Drugs that are both cytotoxic on resting CLL cells and able to inhibit CLLs' activation and subsequent proliferation would be beneficial in the treatment of this disease.

Metformin was first synthesized and found to reduce blood sugar in the 1920s, and is now perhaps the most widely prescribed antidiabetic drug. Recent studies have provided evidence that diabetic patients receiving metformin have a reduced risk of developing cancer and decreased cancer mortality [13, 14]. Although it is not clear yet if these observations apply to non-diabetic populations [15, 16], several studies using tumor cell lines and mouse models established direct actions of metformin on cancer cells (for review [17]). Indeed, metformin reduces tumor growth not only indirectly (systemic effect: glucose and insulin lowering) but also by direct inhibition of energetic metabolism [18] and inhibition of pathways involved in cell proliferation [18–20], through both AMPK-dependent [21, 22] and -independent mechanisms [23–27].

Given these considerations, we studied how metformin interferes with the response of CLL cells to activation stimuli similar to the ones they receive in lymphoid tissues.

We used well-established *in vitro* CLL cell culture systems to recreate a microenvironment where to stimulate quiescent leukemic cells derived ex-vivo from the peripheral blood of CLL patients and drive their proliferation [10]. Accordingly, CLL cells were cultured in the presence of CD40 ligand (CD40L)-expressing mouse fibroblasts, which provide both stromal cell components and T helper signals. CD40L, expressed *in vivo* by CD4 T helper lymphocytes, binds CD40 on the surface of CLL cells and triggers activation pathways [9, 10]. Critical requisite for successful clonal expansion of xenotransplanted CLL cells is indeed the presence of T helper lymphocytes [28]. The *in vitro* cytotoxic and cytostatic effects of metformin on leukemic cells obtained from CLL patients, either quiescent or stimulated to enter the cell cycle, were explored.

## RESULTS

### Metformin affects CLL cell viability, mitochondrial ΔΨ and down-regulates Mcl-1 expression

**Figure 1:**
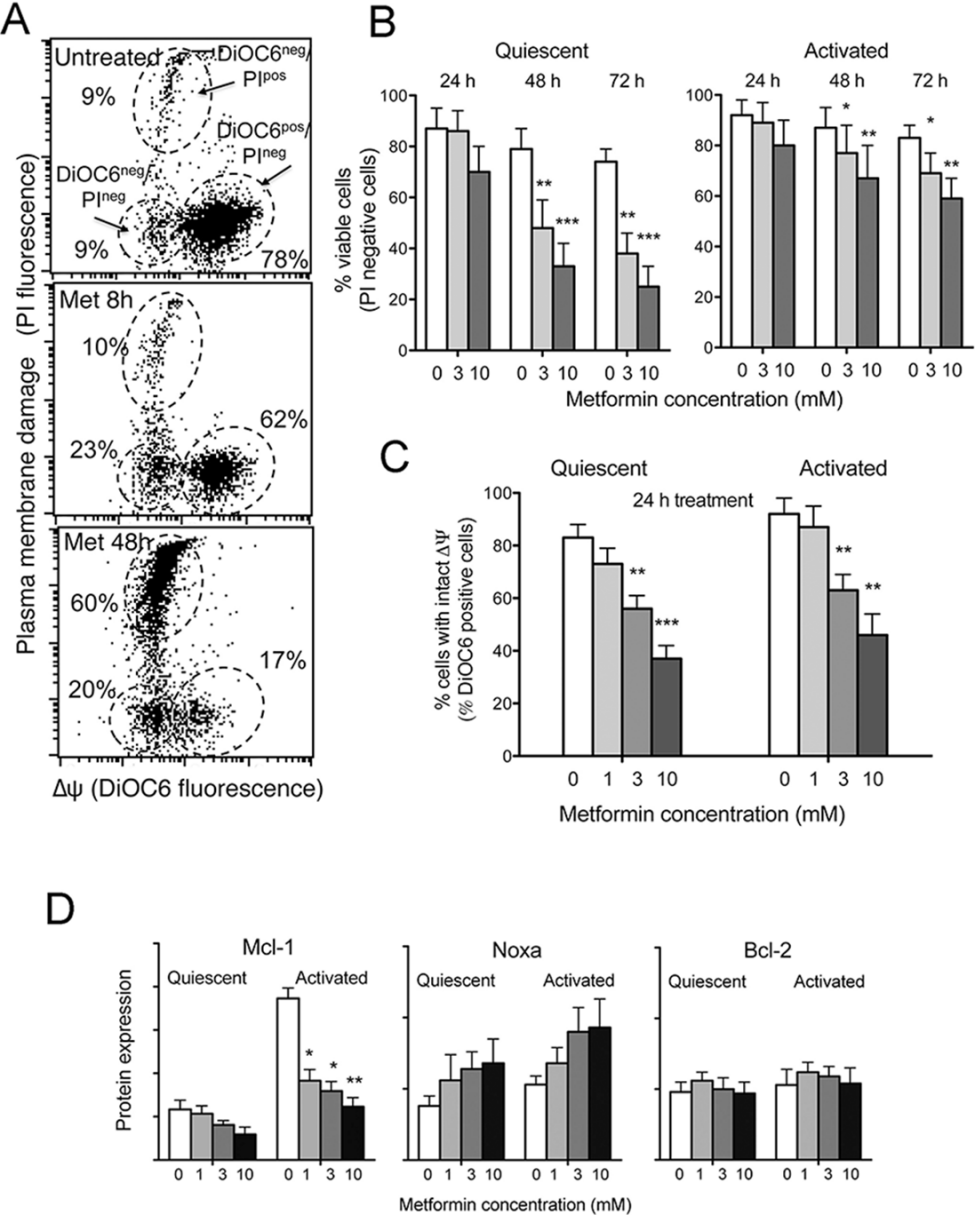
Metformin is cytotoxic to CLL cells, causes ΔΨ dissipation and Mcl-1 down-regulation **A.** Dot plot of DiOC6/PI fluorescence from one CLL sample treated with 3 mM metformin for 48 hours. The following subpopulations are indicated: DiOC6^pos^/PI^neg^ cells (intact ΔΨ), DiOC6^neg^/PI^neg^ cells (dissipated ΔΨ but intact plasma membrane) and DiOC6^neg^/PI^pos^ cells (dead cells). **B.** CLL cell viability in response to metformin treatment at different concentrations, evaluated as % PI-negative cells on DiOC6/PI dot plots. Metformin was added either to unstimulated CLL cells (Quiescent) or CD40L-stimulated (Activated) cells at the beginning of stimulation. Values are the mean on 6 CLL samples (± SD). **C.** Mitochondrial transmembrane potential ΔΨ in response to 24 hours metformin treatment, evaluated as the % of DiOC6-positive cells. The mean of six CLL samples is reported (± SD). **D.** Bcl-2, Mcl-1 and Noxa protein expression measured by flow cytometry on the viable cell subpopulation (‘high-FSC’ cell population in the dot plot, see details in Materials and Methods), of unstimulated (Quiescent) or CD40L-stimulated (Activated) CLL samples treated with metformin. For each CLL sample the geometric mean of protein fluorescence was evaluated for 10.000 cells, and background-deducted. Mean values of 5 CLL samples are shown (± SD).

We first addressed the cytotoxic activity of metformin on CLL cells, both on quiescent and activated CLL cells. Activation was achieved by culturing CLL cells at high cell density and in the presence of CD40L-expressing fibroblasts. The response to CD40L-stimulation was followed by monitoring cellular and molecular parameters associated with CLL activation phenotype (not shown). Metformin was added at the same time of CD40L-fibroblasts. Multiparameter flow cytometry was used to measure cell death (damage of cell plasma membrane by PI uptake) and loss of mitochondrial trans-membrane potential (decreased fluorescence of the cationic voltage dependent fluorescent probe DiOC_6_) (Figure 1A).

We found that metformin exerted dose-dependent cell death, particularly on quiescent CLL cells (Figure 1A and 1B) and ΔΨ dissipation (Figure 1A and 1C). Metformin-induced ΔΨ dissipation was noticed in a substantial fraction of CLL cells already after 8 hours metformin treatment, when no plasma membrane rupture was observed yet (Figure 1A), suggesting that ΔΨ dissipation preceded plasma membrane damage. Drop of mitochondrial transmembrane potential was remarkable in both unstimulated and stimulated CLL culture conditions (Figure 1C), but ended up in frank cell death more effectively in quiescent CLL cells than in activated ones (Figure 1B).

Mcl-1 is one of the most important Bcl-2 family members in CLL cell survival. High levels of this protein have been associated with poor treatment response and high risk of disease progression [29, 30]. We observed by flow cytometric single-cell analysis of Mcl-1 expression that metformin hampered the up-regulation of Mcl-1 occurring in response to CLL activation stimuli (Figure 1D). Mcl-1 is neutralized by Noxa, a proapoptotic member of the Bcl-2 family, and contributes to promote its proteasomal degradation [31]. Metformin induced an increase of Noxa protein expression, as evaluated by flow cytometry, both in quiescent and activated CLL cells (Figure 1D). The expression of Bcl-2 protein remained unchanged (Figure 1D).

Importantly, as protein expression analysis was performed selectively on CLL cells within the flow cytometric ‘high-FSC’ gate, i.e. a gate that contains, in the case of the CLL cell systems, only cells with intact ΔΨ dissipation and plasma membrane (as demonstrated in Bruno et al. [32]), we may deduce that metformin down-regulates Mcl-1 expression before mitochondrial perturbation and cell death.

### Metformin impairs CLL cell activation and cell cycle entry

As mentioned, CLL cells subjected to co-cultures with CD40L-fibroblasts become activated blasts. They progressively increase their size, display an activated phenotype and enter the cell cycle (not shown). It takes about 48 hours after CD40L-stimulation to detect the first CLL cells in S- and G2M-phases of the cell cycle (not shown). Their proportion increases progressively with time (not shown).

**Figure 2:**
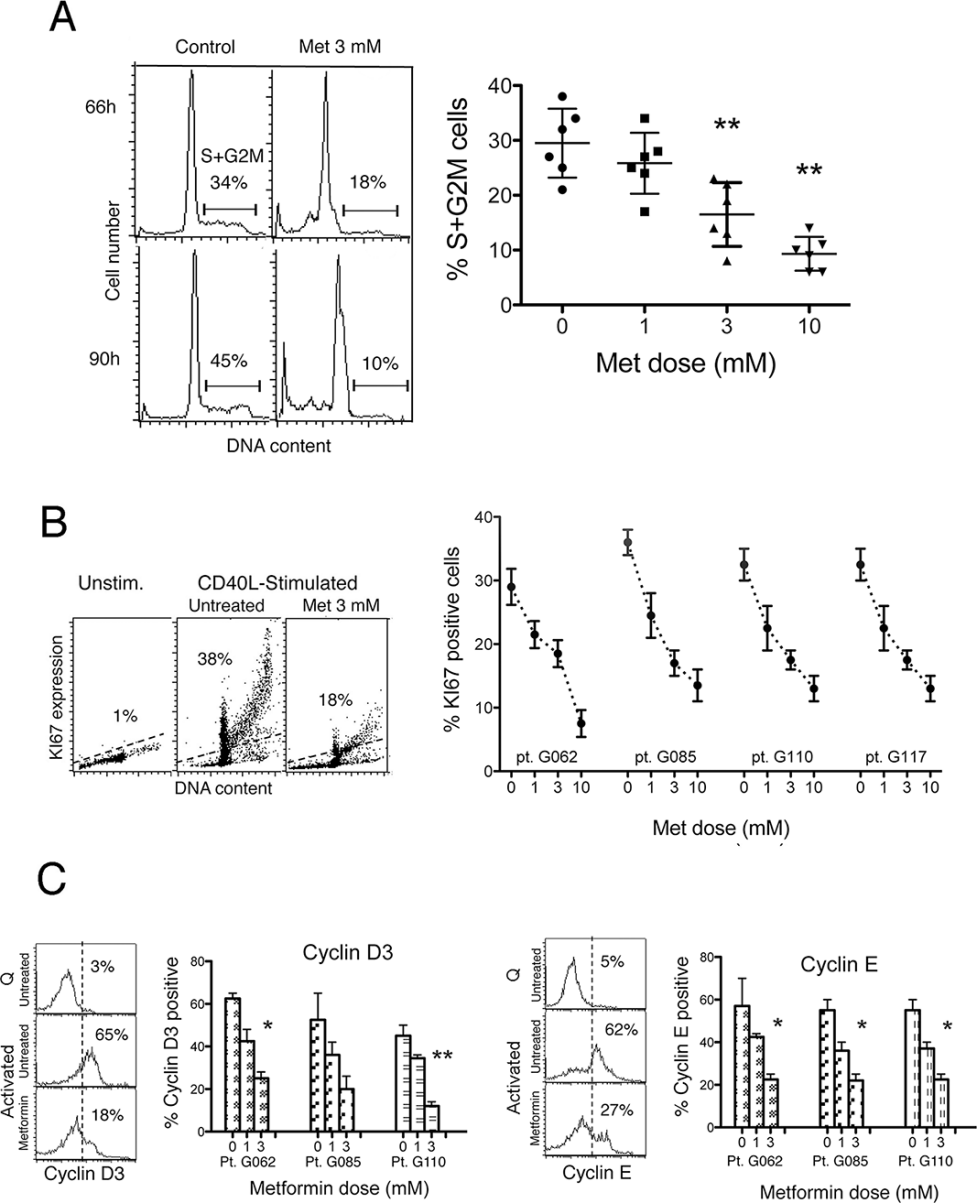
Metformin impairs cell cycle entry of CLL cells **A.** Left: Flow cytometric DNA content histograms of CLL cells cultured with CD40L-fibroblasts for 66 and 90 hours, either in the absence or in the presence of metformin 3 mM. Noticeable, the G0G1 peak is skewed on the right side, likely reflecting an accumulation of CLL cells at the G1-S transition point. The percentage of cells in S+G2M cell cycle phases is reported. Right: Percentage of CLL cells in S+G2M phase in cell cultures of six CLL patients after 66 hours stimulation either in the absence or in the presence of metformin at the doses indicated (Lines are Mean ± SD). **B.** Left: Flow cytometric DNA/KI67 bivariate plots of CLL cells from one patient, un-stimulated or CD40L-stimulated for 66 hours either alone or in the presence of 3 mM metformin. Right: Percentage of Ki-67 positive cells in leukemic cell cultures of four CLL patients, after 66 hours CD40L-stimulation, either untreated or treated with metformin at the doses indicated. **C.** Left-side for each of the two panels: Flow cytometric histograms of cyclin D3 and cyclin E expression of leukemic cells of one CLL patient, un-stimulated (Quiescent, Q) or CD40L-stimulated (Activated) for 66 hours either in the absence or in the presence of 3 mM metformin. Right-side for each of the two panels: Percentage of cyclin D3 and cyclin E positive cells in CLL cultures from three CLL patients after 66 hours CD40L-stimulation, either in the absence or in the presence of metformin at the doses indicated.

We determined whether metformin affected cell cycle entry and mitotic activity. Data from the third and fourth day after stimulation are shown in the flow cytometric DNA content distributions of Figure 2A (left). The rate of increase of the S+G2M cell fraction was diminished when metformin was present in the CLL cell cultures (Figure 2A). The reduction was dose-dependent (Figure 2A right). Likewise, the fraction of Ki-67 positive cells was significantly lower in metformin-treated CLL cells than in untreated controls, in a dose-dependent way (Figure 2B).

The G1-S phase transition is regulated by a tightly tuned interplay between cyclins, serine/threonine cyclin dependent kinases, and cdk inhibitors. D3 and E type cyclins have a key regulatory role in CLL, for the induction of G1 progression and G1/S transition [33, 34]. Therefore, we determined whether metformin-induced impairment of CLL cell cycle entry was associated with inhibition of these cell cycle regulatory proteins. In our CLL samples, metformin dose-dependently reduced the positive effect caused by CD40 ligation on expression of cyclins D3 and E after CD40 ligation (Figure 2C) indicating a direct biguanide interaction with this pathway as a mechanism underlying the decrease in G1-S transition.

Altogether, the data indicate that metformin impairs CLL response to activation stimuli hampering subsequent G1-S transition and clonal expansion.

### Metformin impairs stimulation-induced increase of adhesion and homing molecule expression in CLL cells

The interaction of CLL leukemic cells with the microenvironment plays a pro-activation/proliferation role while altered adhesion properties inhibit their clonal expansion [35].

CD44 promotes CLL disease development and apoptosis-resistance [36]. Adhesion molecules CD54 (ICAM-1) and CD58 (LFA-3) are overexpressed in CLL and are associated with disease progression [37]. CXCR4(CD184) and CD62L mediate CLL cell survival and activation and are involved in homing, being overexpressed by leukemic cells when they are in lymph node and bone marrow [35, 38, 39].

**Figure 3:**
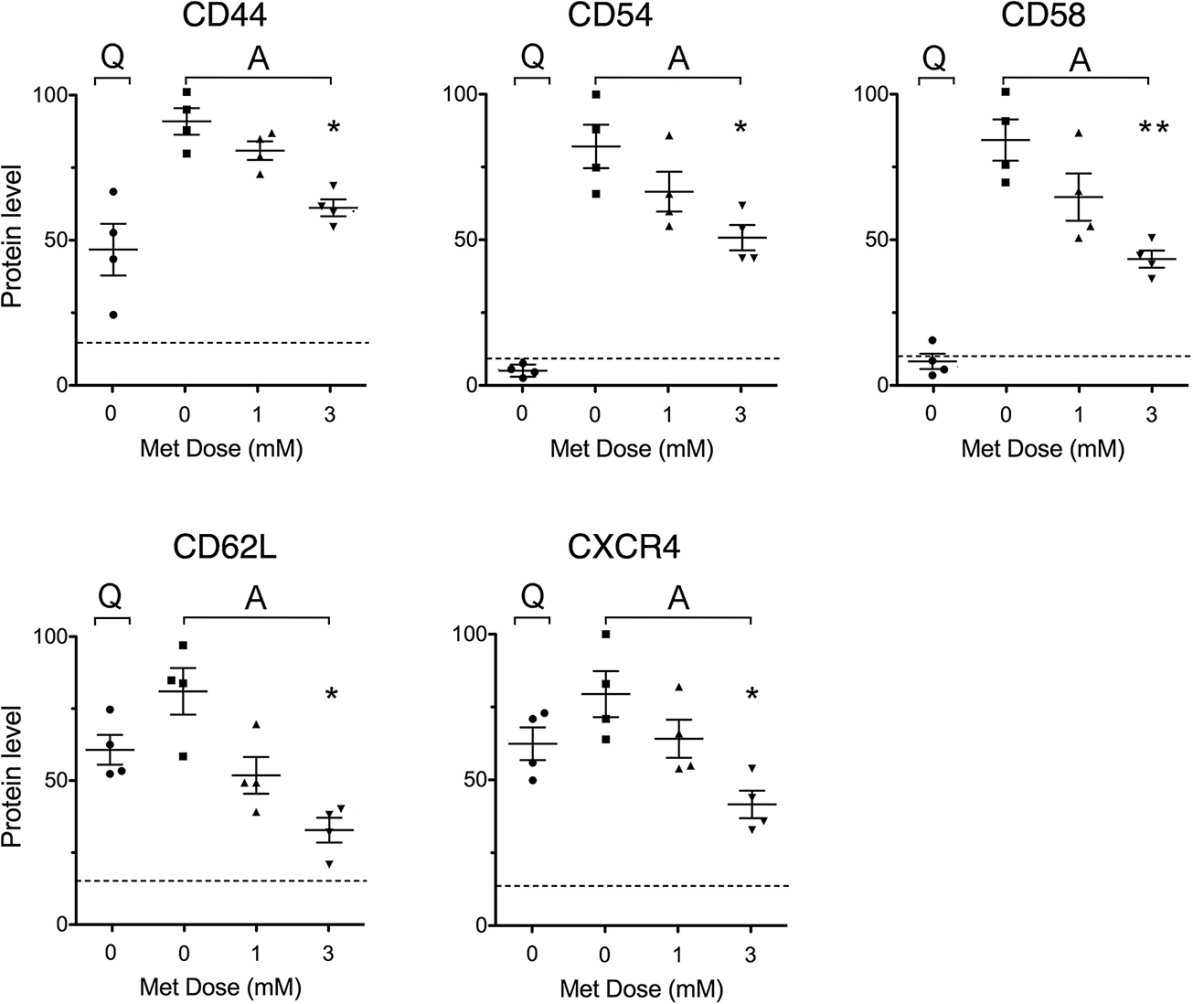
Metformin impairs stimulation-induced up-regulation of adhesion and homing molecule expression Expression of adhesion and homing molecules in leukemic cells from 4 CLL patients, either unstimulated (Quiescent, Q) and untreated, or stimulated with CD40L (Activated, A) and untreated or treated with metformin for 48 hours. For each adhesion/homing molecule, an arbitrary value of 100 was assigned to the CLL sample that displayed the highest expression level. Background levels identified by isotype-matched negative controls are indicated by the dotted line.

Flow cytometric experiments showed that the levels of CD44, CD54 and CD58 were remarkably up-regulated by CD40L-stimulation, as previously described [40, 41]. This increase was significantly and dose-dependently inhibited by metformin (Figure 3). A similar effect was observed for CD62L and CXCR4(CD184) whose response to CD40L-stimulation expectedly showed a dual phase response [42, 43] with an initial drop (in the first 24 hours, not shown) followed by later increase. Again, this response was dose dependently reduced by metformin (Figure 3).

### Metformin impairs stimulation-induced activation of NF-kappaB, STAT3 and Akt in CLL cells

**Figure 4:**
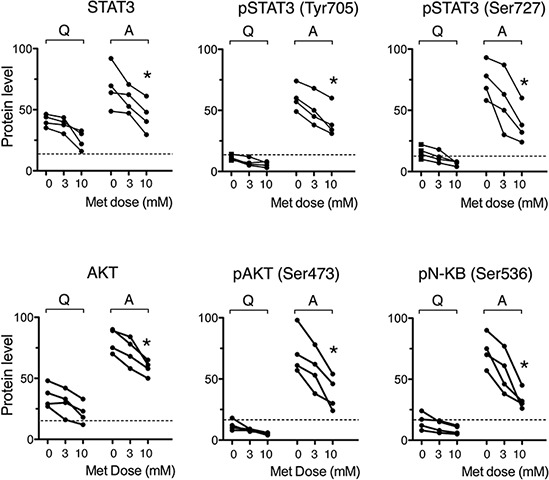
Metformin impairs stimulation-induced up-regulation of NF-kappaB, STAT3 and Akt activity Expression of pan and phosphorylated transcription factors in leukemic cells from 4 CLL patients, quiescent (Q) or stimulated with CD40L (Activated, A) in the absence or presence of metformin for 48 hours. An arbitrary value of 100 was assigned to the CLL sample that displays the highest expression level. Dotted lines indicate the background fluorescence level, as identified by isotype-matched negative controls.

Given that metformin depressed CLL cell cycle progression, we investigated the biguanide effects on related signaling pathways. We addressed PI3/Akt [44–47] and NF-κB/STAT3 [5, 48–51]. These pathways play a major role in CLL pathogenesis. NF-kappaB is up-regulated in CLL cells and sustains CLL cell survival [48–50], proliferation [5], activation-promoting activity of adhesion [51] and homing molecules [52]. Specifically in CLL cells, NF-kappaB is activated by the un-phosphorylated form of STAT-3 [50], which is highly expressed in CLL cells [53]. STAT3 in CLL cells exhibits the peculiarity of being activated not only through the more common tyrosine (Tyr705)-phosphorylation but also through phosphorylation on Ser727 [53, 54]. Pathways involved in CLL prosurvival [44–46] and cell cycle progression [47] signaling are driven also by the serine-threonine kinase Akt. Accordingly, we analyzed the effects of metformin on the intracellular expression of Akt(pan), STAT3(pan) and the phosphorylated forms pSTAT3(Tyr705), pSTAT3(Ser727), pAkt(Ser473), and pNF-kB(Ser536). Quiescent CLL cells did not display significant levels of phosphorylated kinases. CD40L-stimulated CLL cells showed strong up-regulation (Figure 4). The presence of metformin during CD40L-stimulation significantly inhibited the up-regulation of all transcription factors (Figure 4).

### Metformin induces AMPK phosphorylation and reduces glucose metabolism in CLL cells

CLL cells rely on oxidative phosphorylation for their bioenergetics needs [55–57]. One of the best-known mechanisms responsible for cell cycle arrest by metformin is inhibition of mitochondrial respiratory chain complex I [18], which results in ATP depletion [18, 58] and consequent accumulation of AMP, thus inducing phosphorylation of 5′ AMP-activated protein kinase (AMPK) [19].

**Figure 5:**
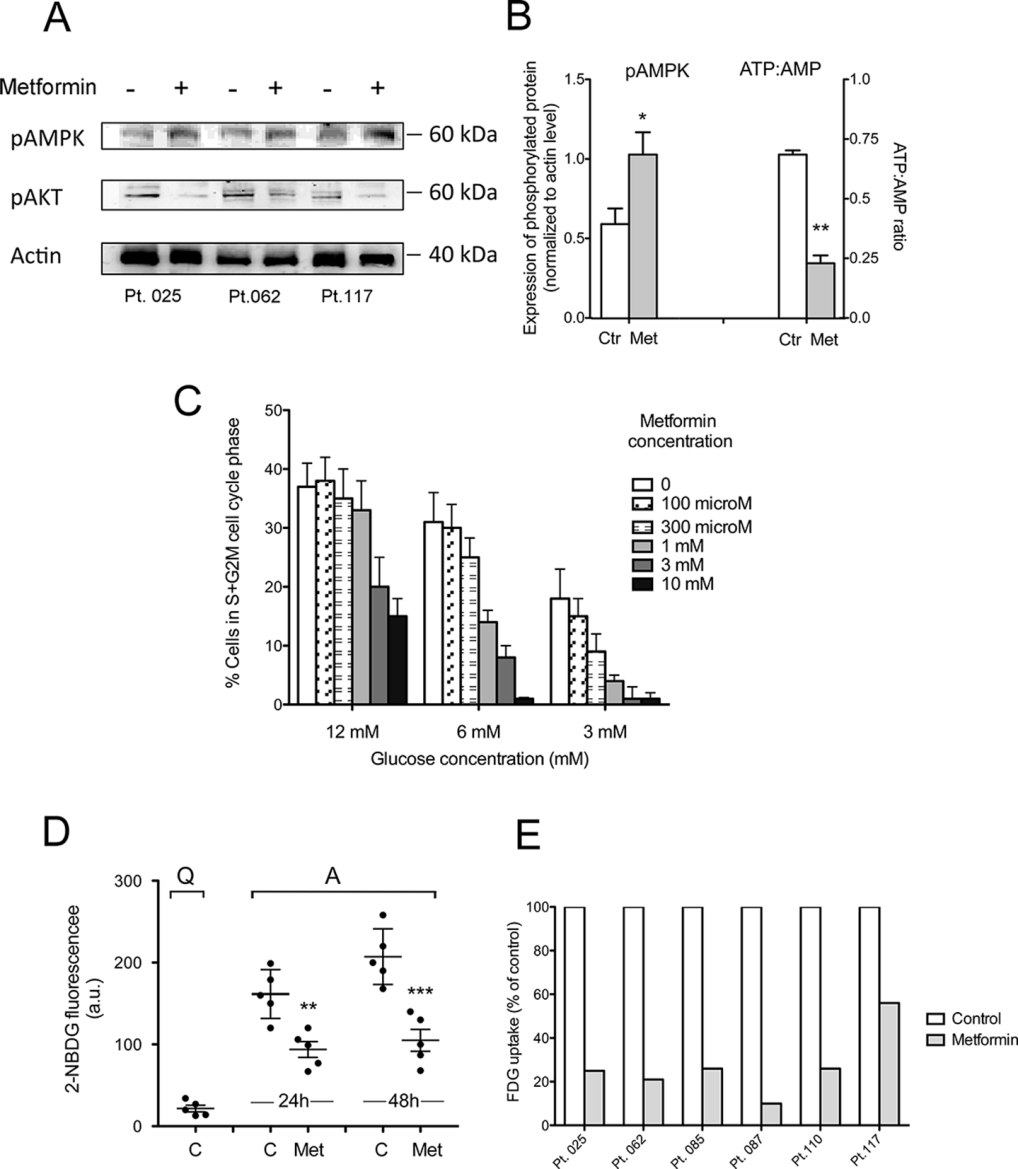
Metformin increases CLL cell AMPK phosphorylation and decreases intracellular phosphorylated glucose available for glycolysis **A.** Western blot of phosphorylated AMPK (*Thr*172) and Akt (*Ser473*) in CD40L-stimulated CLL cells untreated or treated with 10mM metformin. **B.** pAMPK protein expression, as calculated from WB by densitometric analysis and normalized to actin signal and ATP:AMP ratio determined by enzymatic assay (mean ± SE from three CLL patients). **C.** Proliferation of CLL cells cultured in media with different glucose concentrations: standard culture concentration of 12 mM glucose or lowered glucose concentration, i.e. 6 mM and 3 mM. Cells were CD40L-stimulated for 66 hours either in the absence or in the presence of metformin at the doses indicated (range 100 microM–10 mM) and assayed for proliferation by measuring the percentage of cells in S+G2M cell cycle phases, on flow cytometric DNA content histograms. **D.** 2-NBDG fluorescence (arbitrary units) measured by flow cytometry in unstimulated untreated CLL cells (Quiescent, Q), or CD40L-stimulated CLL cells (Activated, A), either untreated or treated with metformin for 48 hours. Results for five CLL patients are shown (mean ± SD). The intracellular glucose available for glycolysis is significantly lower in metformin-treated than in untreated CLL cells. **E.** Metformin-induced reduction of FDG uptake in six samples from 5 CLL patients. FDG uptake is normalized to cell number and expressed as the amount of trapped FDG6P relative to untreated controls (arbitrarily set to 100).

The latter mechanism took place in CLL cells as well. Drug treatment of activated CLL cells remarkably decreased their ATP:AMP ratio (Figure 5B) and increased pAMPK expression (Figure 5A and 5B).

B lymphocytes undergo metabolic reprogramming to aerobic glycolysis (‘Warburg effect’) when they are activated and enter cell proliferation [59]. Mitogenic stimulation is a highly energy demanding process for leukemic cells as well. Accordingly, CLL cells were found to rely on aerobic glycolysis to produce energy for their proliferation [60]. Thus, we expected that lowering extracellular glucose concentration would have enhanced sensitivity to metformin. CLL cells cultured in low glucose medium displayed lower proliferative response to microenvironment stimuli (Figure 5C). When also oxidative phosphorylation was inhibited by metformin, cell cycle block was further enhanced (Figure 5C).

Since AMPK is a negative regulator of the Warburg effect [61], we explored whether metformin affects the glycolytic capacity of activated CLL cells. The novel fluorescent 2-deoxyglucose analog probe 2-NBDG) was used to this purpose. 2-NBDG is transported within the cell by glucose transporters (GLUTs) and is phosphorylated by the same hexokinases responsible for the conversion of glucose into glucose 6-phosphate (G6P) [62]. By the latter reaction the molecule is retained within the cell but, unlike G6P, cannot be further processed [62, 63]. Thus, 2-NBDG fluorescence within the cell tracks the level of intracellular phosphorylated 2-NBDG and provides indirect information on the level of G6P, which lies at the start of fundamental metabolic pathways.

Flow cytometric single-cell data of 2-NBDG fluorescence indicated that the average uptake of 2-NBDG after 48 hours CD40L-stimulation was almost ten fold the uptake of 2-NBDG in quiescent CLL cells (Figure 5D). The presence of metformin during CLL cell activation remarkably inhibited this rise (Figure 5D).

Metformin effect on CLL cell glycolysis was also evaluated by estimating the capability of the cells to retain ^18^F-fluorodeoxyglucose (FDG). This quantitative method confirmed a reduction in overall glucose flux caused by metformin in CLL leukemic cells (Figure 5E).

Taken together, these results indicate that the block in G1-S transition observed when CD40L-stimulated CLL cells are treated with metformin is associated with overall ATP reduction and impairment of the stimulation-induced rise of intracellular glucose phosphorylation and glycolytic ability.

### Combination cytotoxicity of metformin with Fludarabine and ABT-737

We examined the ability of metformin in potentiating *in vitro* cytotoxicity of anti-tumor drugs.

**Figure 6:**
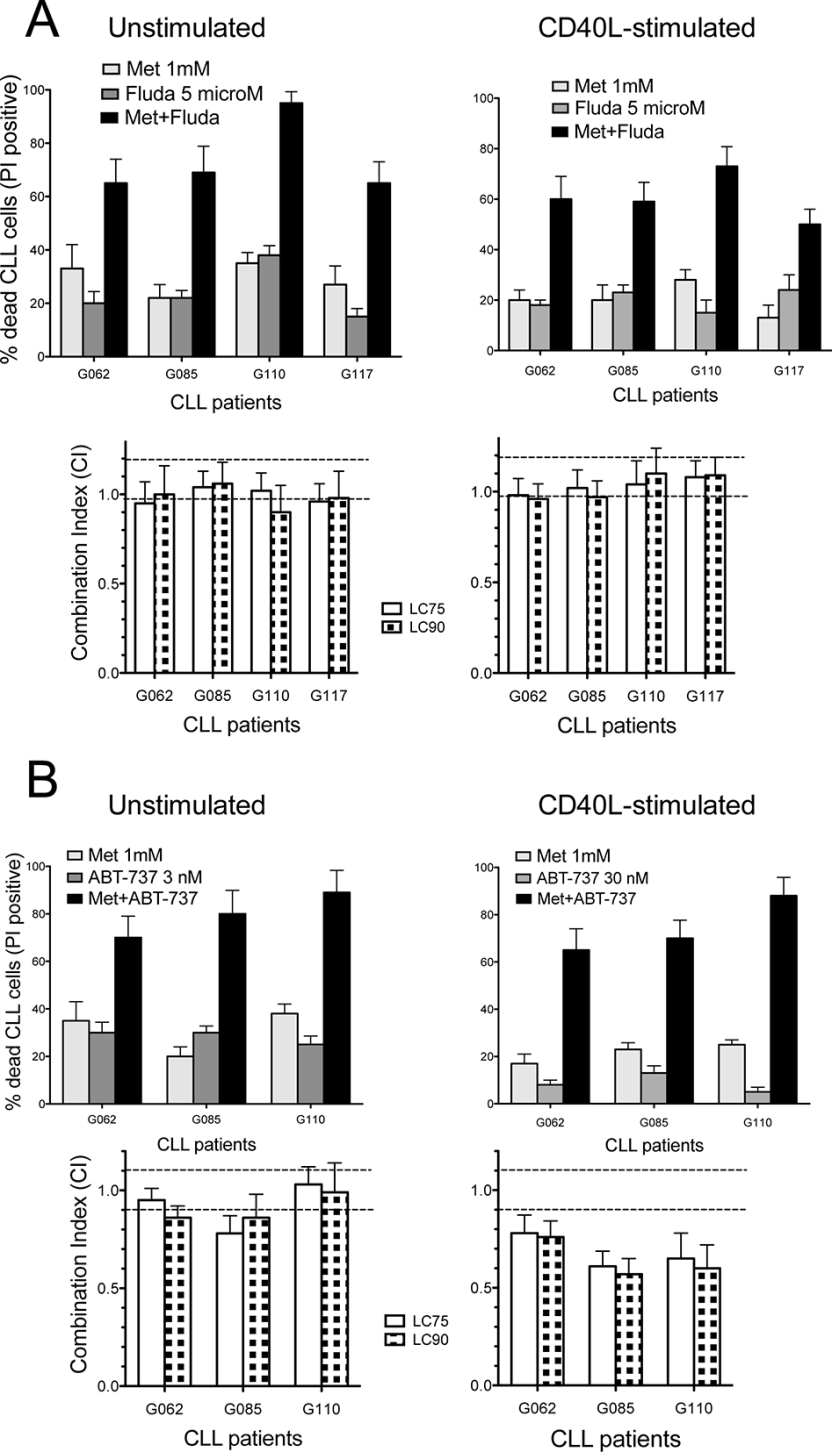
Combination cytotoxicity of metformin and Fludarabine or ABT-737 **A.** Upper: metformin and/or Fludarabine cytotoxicity (48 h) in four CLL samples, resting or activated, expressed as % cells positive for PI uptake (spontaneous apoptosis subtracted). Lower: CI values (at LC75 and LC90, see Materials and Methods) calculated on dose-effect profiles obtained by treating cells with increasing concentrations of metformin (1–10 mM), Fludarabine (1–10 microM) or Met/Fludarabine at constant ratios. Dotted lines separate CI values indicating synergism (CI < 0.9), additive effect (0.9 ≤ CI ≤ 1.1) and antagonism (CI > 1.1). **B.** Upper: metformin and/or ABT-737 cytotoxicity (48 h) in three resting or activated CLL samples. Lower: CI values calculated on dose-effect profiles obtained treating cells with increasing concentrations of metformin (1–10 mM), ABT-737 (1–10 nM for resting CLLs, 10–100 nM for activated samples) or Met/ABT-737 at constant ratios.

Combined treatment of CLL cells with Fludarabine (F-ara-AMP) and metformin induced higher cytotoxicity if compared to each drug alone, in both resting and activated conditions (Figure 6A). Combination Index (CI) data, computed from cytotoxicity profiles of drugs alone or in combination, indicated mostly additivity of the two treatments, and synergy in some cases (Figure 6A).

Co-administration of metformin with the BH3-only mimetic ABT-737 [64] significantly increased the cytotoxicity of sub-lethal doses of ABT-737 (that is, 3 or 30 nM for resting or activated CLLs, respectively) (Figure 6B). Activated/proliferating CLL cells are much more resistant to ABT-737 than quiescent cells are [32, 65]. This is why CD40L-stimulated CLL cells were treated in our dose-response experiments with 10-fold higher doses of ABT-737 compared to quiescent CLL cells. CI values demonstrated a good level of synergy in activated CLL samples, and mostly additive effect in unstimulated ones (Figure 6B).

### CLL cells are more sensitive to metformin than healthy peripheral blood lymphocytes

**Figure 7:**
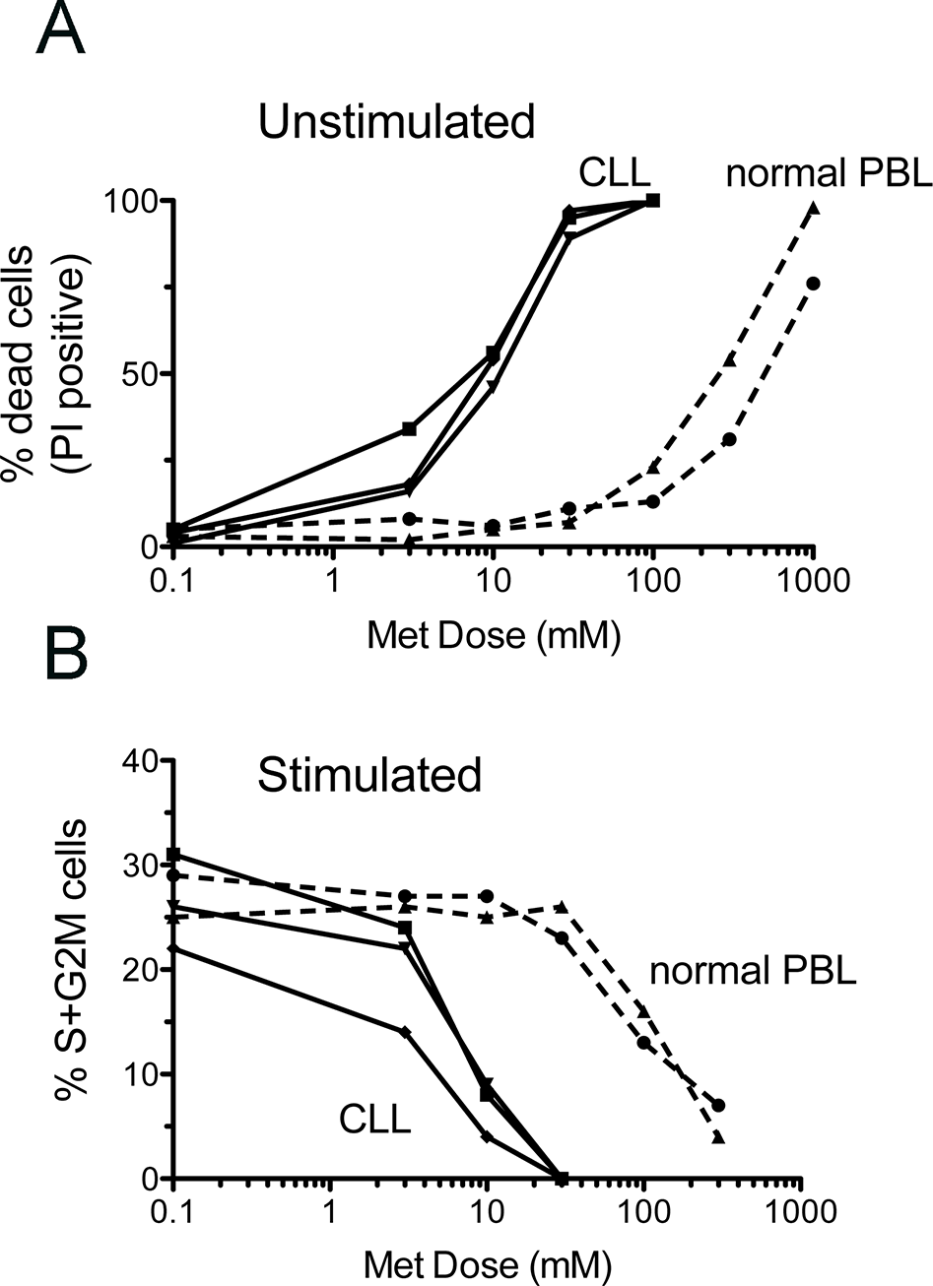
CLL cells are more sensitive to metformin than healthy peripheral blood lymphocytes Peripheral blood lymphocytes (PBL) from two normal donors and leukemic cells from three CLL patients were assayed for survival and proliferation after 66 hours metformin treatment. Upper panel: dose-response curves of the % of dead cells (as evaluated by PI-uptake) in unstimulated PBL and CLL cultures. Lower panel: dose-response curves of the % of cells in S+G2M cell cycle phases in CD40L-stimulated CLL cells and PHA-stimulated normal PBLs.

The cytotoxic action of metformin on peripheral blood lymphocytes from normal donors only occurred at doses significantly higher than those required to affect CLL cell survival (Figure 7A). A similar difference could be documented for the biguanide cytostatic effect that was virtually absent when it was administered on normal lymphocytes at doses that inhibited cell cycle progression of CLL cells (Figure 7B).

## DISCUSSION

In this study we addressed the *in vitro* effects of metformin on CLL cells either quiescent or stimulated to become activated blasts that enter the cell cycle. We found that metformin was cytotoxic to quiescent CLL cells and cytostatic to activated leukemic cells.

Induction of cell death was associated with inhibition of Mcl-1, a Bcl-2 family survival protein highly relevant in CLL [29, 30, 66, 67]. Metformin-mediated down-regulation of this protein did not appear to be only caused by global translational inhibition, as observed for Mcl-1 in metformin-treated head and neck squamous cell carcinoma cell lines [26], since Mcl-1 reduction was associated with increased Noxa expression.

Cytostatic effects of metformin on activated CLL cells were demonstrated by diminished proliferation and diminished expression of proliferation-associated molecules. In these cells metformin inhibited the activation-induced up-regulation of chemokine receptors and adhesion molecules that co-operate to elicit mitotic activity and cell homing in lymphoid tissues, all of which play a pivotal role in the development and progression of CLL [5, 68].

Metformin inhibited the activation of transcription factors lying on CLL pro-survival and pro-activation pathways. Particularly involved in CLL disease progression are PI3/Akt [69] [44–47] and NF-κB/STAT3 [5, 48–51]. According to the results of the present study, metformin exerted an inhibitory effect on all the above pathways.

This inhibitory effect was associated with activation of AMPK and reduction of glucose metabolism. Both mechanisms hamper CLL cell survival, activation and clonal expansion. In particular, AMPK activation opposes tumor progression in several cancer types [70] through inhibition of tumor growth-stimulating mammalian Target of Rapamycin (mTOR) [20], and to down-regulation of cyclins and cyclin dependent kinases (CDKs) to inhibit cell-cycle progression [71]. Reduction of glucose metabolism reduces Mcl-1 protein translation [72] and stabilization [73]. Interestingly, metformin reduced CLL cells glycolytic flux as documented by a reduced capability to retain fluorescent and radioactive probes of sugar metabolism. The well accepted kinetic model of these tracers implies that their phosphorylated forms are false substrates for both phospho-fructoisomerase and glucose-6P-dehydrogenase. Accordingly, the reduction in their cytosol retention caused by metformin has to be considered as a marker of reduced overall glucose flux [74]. In this line, our findings agree with our previous experience in solid tumors and might be at least partially correlated with the interference caused by the biguanide on hexokinase catalytic function [75, 76].

Cytotoxic effects of metformin were previously reported in two studies performed on unstimulated quiescent CLL cells [55, 57]. Both studies attributed this effect on inhibition of oxidative phosphorylation. Here we provide evidence that metformin also modulates glycolytic capability on CLL cells stimulated to enter the cell cycle.

We studied metformin activity in combination with other agents. Cytotoxicity of the classical anti-CLL drug Fludarabine was potentiated by co-administration with metformin. *In vitro* sensitivity of CLL cells to Fludarabine is compromised by abnormal mitochondrial activity, which is typical of CLL cells [56, 77]. Thus, inhibition of mitochondrial respiration exerted by metformin may restore the CLL cell apoptotic response to Fludarabine.

ABT-737 holds promise for the treatment of CLL [64, 78]. However, the cytotoxic activity of this BH3-only mimetic is impaired when Mcl-1 is overexpressed [78] or when cells are activated and proliferate [32, 65]. In our experimental setting metformin potentiated the action of ABT-737 in resting CLL cells, due, at least in part, to its Mcl-1 inhibitory effect. More remarkable was the synergy between metformin and ABT-737 in stimulated cells, possibly because metformin impairs cell cycle progression, thus enhancing ABT-737 cytotoxicity. Co-administration of metformin might increase the cytotoxic activity of drugs that are more effective on non-cycling CLL cells.

Peripheral blood lymphocytes from healthy donors, either quiescent or activated, were insensitive to the cytotoxic or cytostatic action of metformin at the doses that were effective on CLL cells. Leukemic cells rely on ATP availability and glycolysis more than normal cells do. Also, CLL cells are more addicted to the protective activity of Bcl-2 anti-apoptotic proteins compared to normal cells [79]. This may explain, at least in part, the higher metformin-sensitivity of CLL cells compared to normal lymphocytes.

It is important to highlight that the analysis of protein expression by flow cytometry was restricted to viable leukemic cells only (gating out apoptotic cells), thus to CLL cells that have still intact ΔΨ, intact plasma membrane and do not express activated caspase 3 [32]. By this approach early changes of protein expression can be discriminated from changes that are a late consequence of apoptosis.

Metformin is a relatively well-tolerated drug at the typical doses for diabetic patients (1 g, twice daily). At these doses the peak and steady-state plasma concentrations of metformin rarely exceed 50 μM [80, 81], a concentration that is much lower than the doses that were effective in our *in vitro* CLL cell system. However, we found that limiting extracellular glucose availability remarkably increased CLL cell sensitivity to the cytostatic action of metformin. In low-glucose conditions, inhibition of CLL cell cycle entry and proliferation was achieved by metformin doses that were totally ineffective in standard culture conditions. Glucose-starvation or glycolysis inhibition were shown to potentiate metformin cytotoxicity in other tumor cells as well [82] [83, 84] [85]. We could envisage that, within the pseudofollicles of lymphoid tissues where CLL leukemic cells are concentrated and proliferate, glucose may be depleted because of cancer cells' intense use of glycolysis; this would consequently increase leukemic cells' sensitivity to metformin. An additional factor that would increase metformin efficacy in CLL patients treated with standard doses of metformin, resides in the possible accumulation of the drug within the lymphoid tissues where CLL cells are triggered to re-enter the cell cycle. Drug accumulation in concentrations several fold higher than those in blood has been demonstrated in various other tissues [86, 87]. Also, transient high-dose drug exposure could be achieved by nonconventional routes of administration, as recently observed in mice where peak plasma levels of metformin, after intraperitoneal injection, were ~150-fold higher than those obtained with the oral dosing schedule [82, 88].

Both CLL and diabetes mellitus occur more frequently in the elderly. CLL patients under metformin treatment are not rare, considering that half of the diabetic patients take metformin and approximately 20% of CLL patients have diabetes as co-morbidity [89]. However, observation of these patients may not be informative of the real effects of metformin on CLL, for two reasons. First, these patients are not representative of the overall CLL population, as indicated by data from the European Prospective Investigation into Cancer and Nutrition (EPIC) study showing that men with diabetes have double the risk of CLL [90]. Second, most of them were already on metformin when CLL was diagnosed. In these patients CLL developed in the presence of (and maybe ‘despite’) metformin. Hypothesizing a CLL inhibitory role of metformin, one could envisage that, among the cohort of diabetic patients with potential CLL disease (e.g. monoclonal B-cell lymphocytosis (MBL)), the continuous presence of metformin could have selected metformin-resistant forms of MBL progressing to CLL. Instead, metformin-treated diabetic patients with metformin-sensitive MBL cells could have been protected from progression to overt CLL and lost from the cohort of CLL patients. This would introduce a bias and possibly mask the beneficial effects of metformin. Only clinical trials will elucidate whether this inexpensive medication has the potential to become an adjuvant to current CLL therapies. One pilot study of metformin mono-therapy in poor prognosis CLL patients has started in 2012 (NCT 01750567).

## MATERIALS AND METHODS

### CLL cells and drugs

CLL cells were obtained from the peripheral blood of CLL patients, after informed consent according to the Declaration of Helsinki. Mononuclear cells were separated by Ficoll density gradient centrifugation and assayed by flow cytometry (FACSCalibur, BD Biosciences, San Diego, CA) for standard diagnostic immunophenotyping (CD19, CD5, CD23, CD79B, CD22, CD38) and identification of heavy chain (IgM, IgG or, very rarely, IgA) and light chain (kappa or lambda) isotype. Cells were then resuspended in freezing solution (10% DMSO and 90% Fetal Bovine Serum (FBS)) and cryopreserved in liquid nitrogen. Samples from previously untreated CLL patients and containing at least 95% leukemic cells were considered eligible for the study. Clinical and molecular characteristics of the CLL patients utilized in this study are shown as Supplementary Information in Supplementary Table S1.

Cell cultures of CLL cells were performed by seeding thawed cells into RPMI (InVitrogen S.r.l., Milan, Italy) culture medium supplemented with 10% FBS (InVitrogen) at high cell density, 2–4 × 10^6^/ml. Cells were kept in fresh culture medium for three hours before drug addition. *In vitro* activation of CLL cells by CD40L-NIH 3T3 cells was achieved by co-culturing CLL cells in the presence of a stable CD40L-expressing NIH-3T3 murine fibroblast cell line produced in our laboratory, at a cell number ratio 1:100 (fibroblasts:CLL cells). *In vitro* response to CD40L-activation was assessed by increased expression of activation molecules (CD80, CD86, HLA-DR, CD25, CD69, CTLA-4) and the presence of at least 25–30% cells in the S/G2M cell cycle phase three days after co-culture. We did not add cytokines to contribute to CLL sustained proliferation, since the activation level in our culture setting turned out to be adequate enough for the duration of our experiments.

Metformin (1, 1-dimethylbiguanide hydrochloride) was from Sigma-Aldrich (www.sigmaaldrich.com). In drug-combination tests, metformin was added to the cells simultaneously to a second drug, Fludarabine monophosphate from Schering AG (Berlin, Germany) or ABT-737 from Selleck (Houston, TX).

Drug treatment on stimulated CLL cells was performed simultaneously to exposure of CLLs to CD40L-fibroblasts. Freezing/thawing of CLL cells did not influence sensitivity to metformin, since for three CLL patients fresh and frozen/thawed CLL cells were compared, displaying the same level of cytotoxicity.

Cytotoxicity on 3T3-NIH fibroblasts was not observed at the metformin doses that were cytotoxic to CLL cells. Therefore, we could exclude that the observed impairment of cell cycle entry by metformin was due to possible cytotoxicity on the stimulating fibroblasts.

### Flow-cytometric assays for apoptosis and proliferation

Multiparameter flow cytometric analysis of cellular viability by propidium iodide (PI) exclusion assays, mitochondrial transmembrane potential (ΔΨ) by the cationic dye 3, 3′-dihexyloxacarbocyanine iodide (DiOC6) fluorescence and cell cycle-phase distribution by DNA content were previously described [32]. Ki-67 staining procedures are described below.

### Protein expression by flow cytometry and multiparameter analysis

Analysis of protein levels, expressed either on the plasma membrane or intracellularly, was performed by immunofluorescence and flow cytometry on a FACS Calibur (Beckton Dickinson, San Josè, CA, USA).

Antibodies to surface proteins were CD44, CD54 (ICAM-1) and CD58 (LFA-3), CD62L and CXCR4(CD184), all from BD Pharmingen (San Diego, CA). Antibodies to intracellular proteins were rabbit polyclonal anti-Mcl-1 (H-260), anti-cyclin D3 and cyclin E, all from Santa Cruz Biotechnology (San Diego, CA) and used at 20 μg/ml; FITC-conjugated KI67 mAb (BD Pharmingen, San Diego, CA); antibodies specific for transcription factors: Pan-anti-Akt and pan-anti-STAT3 and antibodies to the phosphoproteins pSTAT3(Tyr705), pSTAT3(Ser727), pAkt(Ser473), pNF-kB(Ser536) were all from Cell Signaling Technology, Inc. To optimize antibody-antigen binding cells were fixed with 3% paraformaldehyde and permeabilized with 0.01% Triton X-100 in the case of Mcl-1, cyclins, Akt and Stat3; fixed with 3% paraformaldehyde and permeabilized with ice-cold methanol, 3 minutes, in the case of phospho-proteins; fixed with ice-cold 90% ethanol for KI67. Secondary antibodies, from Molecular Probes (InVitrogen, Eugene, OR), were Goat or Donkey Immunoglobulins, either anti-Rabbit or isotype-specific anti-Mouse, conjugated to Alexa fluorochromes.

For multiparameter analysis purposes, in several cases cells were counterstained with 30 μg/ml PI and 0.5 mg/ml RNase for 30 min at room temperature in the dark, to obtain DNA content distribution of the cell population and, therefore, to assess protein expression in different cell cycle phases.

It is important to highlight that the analysis of protein expression by flow cytometry was restricted to viable leukemic cells, namely cells falling within the ‘live’ gate on flow cytometric FSC-SSC plots, i.e. the ‘high FSC-low SSC’ gate (thus gating out apoptotic cells). This compartment contains CLL cells that have still intact ΔΨ, intact plasma membrane and do not express activated caspase 3 [91]. CLL cells outside the gate, with ‘low FSC-high SSC’ features, are mostly late apoptotic and dead cells, and are not reliable for intracellular immunofluorescence assays because of unspecific staining that easily occurs on dead cells. By this approach early changes of protein expression can be discriminated from consequences of late apoptotic steps.

### ATP and AMP quantification

Cells were washed twice with PBS, lysed with 25% Percloric Acid (PCA) and sonicated in ice three times for 10 seconds. After centrifugation, supernatants containing PCA were collected and neutralized with K_2_CO_3_. ATP and AMP were assayed enzymatically.

ATP was assayed following NADP reduction at 340 nm (Ɛ = 6, 22 mM^−1^ cm^−1^). The medium contained: 50 μg of cells homogenate, 100 mM Tris- HCl pH 8.0, 1 mM NADP, 10 mM MgCl_2_, and 5 mM glucose, 10 IU of purified hexokinase/glucose-6-phosphate dehydrogenase. AMP was assayed following the NADH oxidation at 340 nm (Ɛ = 6, 22 mM^−1^ cm^−1^). The medium contained: 50 μg of cells homogenate, 100 mM Tris-HCl pH 8.0, 75 mM KCl, 5 mM MgCl_2_, 0, 2 mM ATP, 0, 5 mM phosphoenolpyruvate, 0, 2 mM NADH, 10 IU adenylate kinase, 25 IU pyruvate kinase, and 15 IU of lactate dehydrogenase.

### Electrophoresis, semiquantitative western blot (WB), and quantification

The denaturing electrophoresis (SDS–PAGE) was performed using a Laemmli protocol with minor modifications in a Mini Protean III (BioRad, USA) apparatus. Separating gel be a gradient gel from 8% to 14% w/v polyacrylamide, containing 0.1% SDS, at pH 8.8. Stacking gels contained 5% w/v polyacrylamide and 0.1% SDS (at pH 6.8). 20 μg of total proteins were used for each sample. Total protein concentration was estimated by Bradford method. Samples were denaturated by the addition of a solution containing: 8% SDS w/v in 125 mM Tris-HCl (pH 6.8) and 1.25% v/v DTT and incubated for 15 minutes. Then, samples was boiled for 5 min and a second solution, contained 40% w/v sucrose and 0.008% w/v Bromophenol blue was added. Run was performed at 4°C, at 70 mA for each gel, for 120–150 min. Electrophoretically separated samples was transferred onto nitrocellulose (NC) membranes by electroblotting, at 400 mA for 1 h in Tris–glycine buffer (50 mM Tris, 380 mM glycine) plus 20% (v/v) methanol, at 4°C. NC membranes was blocked in 5% Bovine serum Albumine (BSA) overnight, then washed in 0.15% Phoshate Buffered Saline-Tween (PBS-T) and incubated with the specific antibodies (Abs) (o.n at 4°C): rabbit polyclonal anti-phospho AMPK (Cell Signaling Technology, USA, #2531, diluted 1:1000 in PBS-T); rabbit polyclonal anti- phospho AKT (Cell Signaling Technology, USA, #4060, diluted 1:1000 in PBSt); goat polyclonal anti-actin (Santa Cruz Inc. USA, sc-1616, diluted 1:800 in PBS-T). Secondary HPR-conjugated Ab_s_ were diluted in PBS at 1:10000. After extensive washing with 0.15% PBST, binding of Abs was revealed by enhanced chemiluminescence detection system. Blots were acquired by ChemiDoc (BioRad, Hercules, CA, USA). Each band was converted by ChemiDoc into a densitometric trace allowing calculations of intensity and signals normalized on the signal of actin, used as the housekeeping protein.

### 2-NBDG uptake

The fluorescent 2-deoxyglucose analog probe 2-[*N*-(7-nitrobenz-2-oxa-1, 3-diazol-4-yl)amino]-2-deoxyglucose (2-NBDG) [62] was administered to live cells for 15 minutes at the concentration of 50 microM in glucose-free medium. Cells were washed three times with PBS and fluorescence of the probe assayed by flow cytometry (FACS Calibur, Beckton Dickinson) on the FL1 detection channel (515–545 nm). For each CLL sample the geometric mean of the fluorescence intensity of 10.000 CLL cells was calculated.

### FDG uptake

Labelling was performed by incubating 2 × 10^6^ CLL cells with FDG, as described [76]. Glucose-free medium was used for FDG uptake and added with PBS and FDG so to obtain a tracer concentration of 1 microCi/mL. Tracer exposure was maintained for 60 minutes at 37°C. Cells then rinsed twice in cold PBS, and counted to determine cell associated fluorine-18 radioactivity, using a Packard Cobra II gamma counter (Packard, Meriden, CT). FDG retention was measured as the ratio between bound and total radioactivity. Washing did not induce significant efflux of FDG, as confirmed by preliminary studies comparing FDG uptake of cells washed for different periods of time in the presence or absence of the glucose transport inhibitors, cytochalasin B and phloretin. This indicates that after an incubation period of 60 minutes, almost all FDG was trapped intra cells as FDG6P. Radioactivity uptake values were normalized to the number of viable (trypan blue-negative) cells.

### Combination cytotoxicity and statistics

Combined cytotoxicity of metformin administered together with Fludarabine or ABT-737 was calculated by the Chou–Talalay method [92] with the CalcuSyn software (Biosoft, Cambridge, UK). Combination index (CI) was computed from dose–effect curves of drugs alone and in combination. CI represents a measure of the effect of drug interaction (additive effect: 0.9 ≤ CI ≤ 1.1, synergism: CI < 0.9 and antagonism: CI > 1.1). It depends on the ‘fractional effect level’ at which it is calculated. We considered two levels of cytotoxicity, LC75 and LC90 (concentration lethal to 75% and 90% of CLL cells). Drugs were always combined at constant molar ratios. Met:ABT-737 ratios were 10^6^:1 for resting CLL samples and 10^5^:1 for CD40L-stimulated samples (as activation augmented ABT-737 chemoresistance). Met:Fludara ratio was 200:1.

For statistical comparison between samples, the Mann-Whitney *U* test was used for unpaired sample data. Paired sample data were analyzed by the paired Student's *t*-test or Wilcoxon signed-rank test. Analyses were performed using the GraphPad Prism version 5.00 statistical software (GraphPad Software Inc., La Jolla, CA, USA). Figure notations: *p ≤ 0.05; **p ≤ 0.01; ***p ≤ 0.001; n.s., not significant.

## SUPPLEMENTARY TABLES



## References

[1] Rosenwald A, Alizadeh AA, Widhopf G, Simon R, Davis RE, Yu X, Yang L, Pickeral OK, Rassenti LZ, Powell J, Botstein D, Byrd JC, Grever MR, Cheson BD, Chiorazzi N, Wilson WH (2001). Relation of gene expression phenotype to immunoglobulin mutation genotype in B cell chronic lymphocytic leukemia. J Exp Med.

[2] Messmer BT, Messmer D, Allen SL, Kolitz JE, Kudalkar P, Cesar D, Murphy EJ, Koduru P, Ferrarini M, Zupo S, Cutrona G, Damle RN, Wasil T, Rai KR, Hellerstein MK, Chiorazzi N (2005). *In vivo* measurements document the dynamic cellular kinetics of chronic lymphocytic leukemia B cells. J Clin Invest.

[3] Calissano C, Damle RN, Hayes G, Murphy EJ, Hellerstein MK, Moreno C, Sison C, Kaufman MS, Kolitz JE, Allen SL, Rai KR, Chiorazzi N (2009). *In vivo* intraclonal and interclonal kinetic heterogeneity in B-cell chronic lymphocytic leukemia. Blood.

[4] Quijano S, Lopez A, Rasillo A, Barrena S, Luz Sanchez M, Flores J, Fernandez C, Sayagues JM, Osuna CS, Fernandez N, Gonzalez M, Giraldo P, Giralt M, Perez MC, Martin-Antoran JM, Gutierrez O (2008). Association between the proliferative rate of neoplastic B cells, their maturation stage, and underlying cytogenetic abnormalities in B-cell chronic lymphoproliferative disorders: analysis of a series of 432 patients. Blood.

[5] Herishanu Y, Perez-Galan P, Liu D, Biancotto A, Pittaluga S, Vire B, Gibellini F, Njuguna N, Lee E, Stennett L, Raghavachari N, Liu P, McCoy JP, Raffeld M, Stetler-Stevenson M, Yuan C (2011). The lymph node microenvironment promotes B-cell receptor signaling, NF-kappaB activation, and tumor proliferation in chronic lymphocytic leukemia. Blood.

[6] Pepper C, Ward R, Lin TT, Brennan P, Starczynski J, Musson M, Rowntree C, Bentley P, Mills K, Pratt G, Fegan C (2007). Highly purified CD38+ and CD38- sub-clones derived from the same chronic lymphocytic leukemia patient have distinct gene expression signatures despite their monoclonal origin. Leukemia.

[7] Calissano C, Damle RN, Marsilio S, Yan XJ, Yancopoulos S, Hayes G, Emson C, Murphy EJ, Hellerstein MK, Sison C, Kaufman MS, Kolitz JE, Allen SL, Rai KR, Ivanovic I, Dozmorov IM (2011). Intraclonal complexity in chronic lymphocytic leukemia: fractions enriched in recently born/divided and older/quiescent cells. Mol Med.

[8] Coelho V, Krysov S, Steele A, Sanchez Hidalgo M, Johnson PW, Chana PS, Packham G, Stevenson FK, Forconi F (2013). Identification in CLL of circulating intraclonal subgroups with varying B-cell receptor expression and function. Blood.

[9] Banchereau J, Rousset F (1991). Growing human B lymphocytes in the CD40 system. Nature.

[10] Willimott S, Baou M, Huf S, Wagner SD (2007). Separate cell culture conditions to promote proliferation or quiescent cell survival in chronic lymphocytic leukemia. Leuk Lymphoma.

[11] Shanafelt TD, Jelinek D, Tschumper R, Schwager S, Nowakowski G, DeWald GW, Kay NE (2006). Cytogenetic abnormalities can change during the course of the disease process in chronic lymphocytic leukemia. J Clin Oncol.

[12] Krober A, Bloehdorn J, Hafner S, Buhler A, Seiler T, Kienle D, Winkler D, Bangerter M, Schlenk RF, Benner A, Lichter P, Dohner H, Stilgenbauer S (2006). Additional genetic high-risk features such as 11q deletion, 17p deletion, and V3–21 usage characterize discordance of ZAP-70 and VH mutation status in chronic lymphocytic leukemia. J Clin Oncol.

[13] Zhang P, Li H, Tan X, Chen L, Wang S (2013). Association of metformin use with cancer incidence and mortality: a meta-analysis. Cancer Epidemiol.

[14] Currie CJ, Poole CD, Jenkins-Jones S, Gale EA, Johnson JA, Morgan CL (2012). Mortality after incident cancer in people with and without type 2 diabetes: impact of metformin on survival. Diabetes Care.

[15] Gandini S, Puntoni M, Heckman-Stoddard BM, Dunn BK, Ford L, DeCensi A, Szabo E (2014). Metformin and Cancer Risk and Mortality: A Systematic Review and Meta-Analysis taking into account Biases and Confounders. Cancer Prev Res (Phila).

[16] Bonanni B, Puntoni M, Cazzaniga M, Pruneri G, Serrano D, Guerrieri-Gonzaga A, Gennari A, Trabacca MS, Galimberti V, Veronesi P, Johansson H, Aristarco V, Bassi F, Luini A, Lazzeroni M, Varricchio C (2012). Dual effect of metformin on breast cancer proliferation in a randomized presurgical trial. J Clin Oncol.

[17] Pollak M (2013). Potential applications for biguanides in oncology. J Clin Invest.

[18] El-Mir MY, Nogueira V, Fontaine E, Averet N, Rigoulet M, Leverve X (2000). Dimethylbiguanide inhibits cell respiration via an indirect effect targeted on the respiratory chain complex I. J Biol Chem.

[19] Zhou G, Myers R, Li Y, Chen Y, Shen X, Fenyk-Melody J, Wu M, Ventre J, Doebber T, Fujii N, Musi N, Hirshman MF, Goodyear LJ, Moller DE (2001). Role of AMP-activated protein kinase in mechanism of metformin action. J Clin Invest.

[20] Zoncu R, Efeyan A, Sabatini DM (2011). mTOR: from growth signal integration to cancer, diabetes and ageing. Nat Rev Mol Cell Biol.

[21] Zordoky BN, Bark D, Soltys CL, Sung MM, Dyck JR (2014). The anti-proliferative effect of metformin in triple- negative MDA-MB-231 breast cancer cells is highly dependent on glucose concentration: implications for cancer therapy and prevention. Biochim Biophys Acta.

[22] Cantrell LA, Zhou C, Mendivil A, Malloy KM, Gehrig PA, Bae-Jump VL (2010). Metformin is a potent inhibitor of endometrial cancer cell proliferation—implications for a novel treatment strategy. Gynecol Oncol.

[23] Wurth R, Pattarozzi A, Gatti M, Bajetto A, Corsaro A, Parodi A, Sirito R, Massollo M, Marini C, Zona G, Fenoglio D, Sambuceti G, Filaci G, Daga A, Barbieri F, Florio T (2013). Metformin selectively affects human glioblastoma tumor-initiating cell viability: A role for metformin-induced inhibition of Akt. Cell Cycle.

[24] Ben Sahra I, Regazzetti C, Robert G, Laurent K, Le Marchand-Brustel Y, Auberger P, Tanti JF, Giorgetti-Peraldi S, Bost F (2011). Metformin, independent of AMPK, induces mTOR inhibition and cell-cycle arrest through REDD1. Cancer Res.

[25] Memmott RM, Mercado JR, Maier CR, Kawabata S, Fox SD, Dennis PA (2010). Metformin prevents tobacco carcinogen—induced lung tumorigenesis. Cancer Prev Res (Phila).

[26] Sikka A, Kaur M, Agarwal C, Deep G, Agarwal R (2012). Metformin suppresses growth of human head and neck squamous cell carcinoma via global inhibition of protein translation. Cell Cycle.

[27] Gritti M, Wurth R, Angelini M, Barbieri F, Peretti M, Pizzi E, Pattarozzi A, Carra E, Sirito R, Daga A, Curmi PM, Mazzanti M, Florio T (2014). Metformin repositioning as antitumoral agent: selective antiproliferative effects in human glioblastoma stem cells, via inhibition of CLIC1-mediated ion current. Oncotarget.

[28] Bagnara D, Kaufman MS, Calissano C, Marsilio S, Patten PE, Simone R, Chum P, Yan XJ, Allen SL, Kolitz JE, Baskar S, Rader C, Mellstedt H, Rabbani H, Lee A, Gregersen PK (2011). A novel adoptive transfer model of chronic lymphocytic leukemia suggests a key role for T lymphocytes in the disease. Blood.

[29] Pepper C, Lin TT, Pratt G, Hewamana S, Brennan P, Hiller L, Hills R, Ward R, Starczynski J, Austen B, Hooper L, Stankovic T, Fegan C (2008). Mcl-1 expression has *in vitro* and *in vivo* significance in chronic lymphocytic leukemia and is associated with other poor prognostic markers. Blood.

[30] Veronese L, Tournilhac O, Verrelle P, Davi F, Dighiero G, Chautard E, Veyrat-Masson R, Kwiatkowski F, Goumy C, Vago P, Travade P, Tchirkov A (2008). Low MCL-1 mRNA expression correlates with prolonged survival in B-cell chronic lymphocytic leukemia. Leukemia.

[31] Certo M, Del Gaizo Moore V, Nishino M, Wei G, Korsmeyer S, Armstrong SA, Letai A (2006). Mitochondria primed by death signals determine cellular addiction to antiapoptotic BCL-2 family members. Cancer Cell.

[32] Bruno S, Ghiotto F, Tenca C, Mazzarello AN, Bono M, Luzzi P, Casciaro S, Recchia A, Decensi A, Morabito F, Fais F (2012). N-(4-hydroxyphenyl)retinamide promotes apoptosis of resting and proliferating B-cell chronic lymphocytic leukemia cells and potentiates fludarabine and ABT-737 cytotoxicity. Leukemia.

[33] Decker T, Schneller F, Hipp S, Miething C, Jahn T, Duyster J, Peschel C (2002). Cell cycle progression of chronic lymphocytic leukemia cells is controlled by cyclin D2, cyclin D3, cyclin-dependent kinase (cdk) 4 and the cdk inhibitor p27. Leukemia.

[34] Sherr CJ (1996). Cancer cell cycles. Science.

[35] Burger JA, Peled A (2009). CXCR4 antagonists: targeting the microenvironment in leukemia and other cancers. Leukemia.

[36] Fedorchenko O, Stiefelhagen M, Peer-Zada AA, Barthel R, Mayer P, Eckei L, Breuer A, Crispatzu G, Rosen N, Landwehr T, Lilienthal N, Mollmann M, Montesinos-Rongen M, Heukamp L, Durig J, Hallek M (2013). CD44 regulates the apoptotic response and promotes disease development in chronic lymphocytic leukemia. Blood.

[37] Kimby E, Rincon J, Patarroyo M, Mellstedt H (1994). Expression of adhesion molecules CD11/CD18 (Leu-CAMs, beta 2-integrins), CD54 (ICAM-1) and CD58 (LFA-3) in B-chronic lymphocytic leukemia. Leuk Lymphoma.

[38] Giannoni P, Scaglione S, Quarto R, Narcisi R, Parodi M, Balleari E, Barbieri F, Pattarozzi A, Florio T, Ferrini S, Corte G, de Totero D (2011). An interaction between hepatocyte growth factor and its receptor (c-MET) prolongs the survival of chronic lymphocytic leukemic cells through STAT3 phosphorylation: a potential role of mesenchymal cells in the disease. Haematologica.

[39] Burgess M, Gill D, Singhania R, Cheung C, Chambers L, Renyolds BA, Smith L, Mollee P, Saunders N, McMillan NA (2013). CD62L as a therapeutic target in chronic lymphocytic leukemia. Clin Cancer Res.

[40] Girbl T, Hinterseer E, Grossinger EM, Asslaber D, Oberascher K, Weiss L, Hauser-Kronberger C, Neureiter D, Kerschbaum H, Naor D, Alon R, Greil R, Hartmann TN (2013). CD40-mediated activation of chronic lymphocytic leukemia cells promotes their CD44-dependent adhesion to hyaluronan and restricts CCL21-induced motility. Cancer Res.

[41] Van den Hove LE, Van Gool SW, Vandenberghe P, Bakkus M, Thielemans K, Boogaerts MA, Ceuppens JL (1997). CD40 triggering of chronic lymphocytic leukemia B cells results in efficient alloantigen presentation and cytotoxic T lymphocyte induction by up-regulation of CD80 and CD86 costimulatory molecules. Leukemia.

[42] Vlad A, Deglesne PA, Letestu R, Saint-Georges S, Chevallier N, Baran-Marszak F, Varin-Blank N, Ajchenbaum-Cymbalista F, Ledoux D (2009). Down-regulation of CXCR4 and CD62L in chronic lymphocytic leukemia cells is triggered by B-cell receptor ligation and associated with progressive disease. Cancer Res.

[43] Quiroga MP, Balakrishnan K, Kurtova AV, Sivina M, Keating MJ, Wierda WG, Gandhi V, Burger JA (2009). B-cell antigen receptor signaling enhances chronic lymphocytic leukemia cell migration and survival: specific targeting with a novel spleen tyrosine kinase inhibitor, R406. Blood.

[44] Hofbauer SW, Pinon JD, Brachtl G, Haginger L, Wang W, Johrer K, Tinhofer I, Hartmann TN, Greil R (2010). Modifying akt signaling in B-cell chronic lymphocytic leukemia cells. Cancer Res.

[45] Zhuang J, Hawkins SF, Glenn MA, Lin K, Johnson GG, Carter A, Cawley JC, Pettitt AR (2010). Akt is activated in chronic lymphocytic leukemia cells and delivers a pro-survival signal: the therapeutic potential of Akt inhibition. Haematologica.

[46] Longo PG, Laurenti L, Gobessi S, Sica S, Leone G, Efremov DG (2008). The Akt/Mcl-1 pathway plays a prominent role in mediating antiapoptotic signals downstream of the B-cell receptor in chronic lymphocytic leukemia B cells. Blood.

[47] Longo PG, Laurenti L, Gobessi S, Petlickovski A, Pelosi M, Chiusolo P, Sica S, Leone G, Efremov DG (2007). The Akt signaling pathway determines the different proliferative capacity of chronic lymphocytic leukemia B-cells from patients with progressive and stable disease. Leukemia.

[48] Cuni S, Perez-Aciego P, Perez-Chacon G, Vargas JA, Sanchez A, Martin-Saavedra FM, Ballester S, Garcia-Marco J, Jorda J, Durantez A (2004). A sustained activation of PI3K/NF-kappaB pathway is critical for the survival of chronic lymphocytic leukemia B cells. Leukemia.

[49] Hewamana S, Alghazal S, Lin TT, Clement M, Jenkins C, Guzman ML, Jordan CT, Neelakantan S, Crooks PA, Burnett AK, Pratt G, Fegan C, Rowntree C, Brennan P, Pepper C (2008). The NF-kappaB subunit Rel A is associated with *in vitro* survival and clinical disease progression in chronic lymphocytic leukemia and represents a promising therapeutic target. Blood.

[50] Liu Z, Hazan-Halevy I, Harris DM, Li P, Ferrajoli A, Faderl S, Keating MJ, Estrov Z (2011). STAT-3 activates NF-kappaB in chronic lymphocytic leukemia cells. Mol Cancer Res.

[51] Kesanakurti D, Chetty C, Rajasekhar Maddirela D, Gujrati M, Rao JS (2013). Essential role of cooperative NF-kappaB and Stat3 recruitment to ICAM-1 intronic consensus elements in the regulation of radiation-induced invasion and migration in glioma. Oncogene.

[52] Kukreja P, Abdel-Mageed AB, Mondal D, Liu K, Agrawal KC (2005). Up-regulation of CXCR4 expression in PC-3 cells by stromal-derived factor-1alpha (CXCL12) increases endothelial adhesion and transendothelial migration: role of MEK/ERK signaling pathway-dependent NF-kappaB activation. Cancer Res.

[53] Hazan-Halevy I, Harris D, Liu Z, Liu J, Li P, Chen X, Shanker S, Ferrajoli A, Keating MJ, Estrov Z (2010). STAT3 is constitutively phosphorylated on serine 727 residues, binds DNA, and activates transcription in CLL cells. Blood.

[54] Lee YK, Shanafelt TD, Bone ND, Strege AK, Jelinek DF, Kay NE (2005). VEGF receptors on chronic lymphocytic leukemia (CLL) B cells interact with STAT 1 and 3: implication for apoptosis resistance. Leukemia.

[55] Martinez Marignac VL, Smith S, Toban N, Bazile M, Aloyz R (2013). Resistance to Dasatinib in primary chronic lymphocytic leukemia lymphocytes involves AMPK-mediated energetic re-programming. Oncotarget.

[56] Jitschin R, Hofmann AD, Bruns H, Giessl A, Bricks J, Berger J, Saul D, Eckart MJ, Mackensen A, Mougiakakos D (2014). Mitochondrial metabolism contributes to oxidative stress and reveals therapeutic targets in chronic lymphocytic leukemia. Blood.

[57] Adekola KU, Aydemir SD, Ma S, Zhou Z, Rosen ST, Shanmugam M (2014). Investigating and Targeting Chronic Lymphocytic Leukemia Metabolism with the HIV Protease Inhibitor Ritonavir and Metformin. Leuk Lymphoma.

[58] Owen MR, Doran E, Halestrap AP (2000). Evidence that metformin exerts its anti-diabetic effects through inhibition of complex 1 of the mitochondrial respiratory chain. Biochem J.

[59] Altman BJ, Dang CV (2012). Normal and cancer cell metabolism: lymphocytes and lymphoma. FEBS J.

[60] Tili E, Michaille JJ, Luo Z, Volinia S, Rassenti LZ, Kipps TJ, Croce CM (2012). The down-regulation of miR-125b in chronic lymphocytic leukemias leads to metabolic adaptation of cells to a transformed state. Blood.

[61] Faubert B, Boily G, Izreig S, Griss T, Samborska B, Dong Z, Dupuy F, Chambers C, Fuerth BJ, Viollet B, Mamer OA, Avizonis D, DeBerardinis RJ, Siegel PM, Jones RG (2013). AMPK is a negative regulator of the Warburg effect and suppresses tumor growth *in vivo*. Cell Metab.

[62] Yoshioka K, Takahashi H, Homma T, Saito M, Oh KB, Nemoto Y, Matsuoka H (1996). A novel fluorescent derivative of glucose applicable to the assessment of glucose uptake activity of Escherichia coli. Biochim Biophys Acta.

[63] Zou C, Wang Y, Shen Z (2005). 2-NBDG as a fluorescent indicator for direct glucose uptake measurement. J Biochem Biophys Methods.

[64] Oltersdorf T, Elmore SW, Shoemaker AR, Armstrong RC, Augeri DJ, Belli BA, Bruncko M, Deckwerth TL, Dinges J, Hajduk PJ, Joseph MK, Kitada S, Korsmeyer SJ, Kunzer AR, Letai A, Li C (2005). An inhibitor of Bcl-2 family proteins induces regression of solid tumours. Nature.

[65] Vogler M, Butterworth M, Majid A, Walewska RJ, Sun XM, Dyer MJ, Cohen GM (2009). Concurrent up-regulation of BCL-XL and BCL2A1 induces approximately 1000-fold resistance to ABT-737 in chronic lymphocytic leukemia. Blood.

[66] Smit LA, Hallaert DY, Spijker R, de Goeij B, Jaspers A, Kater AP, van Oers MH, van Noesel CJ, Eldering E (2007). Differential Noxa/Mcl-1 balance in peripheral versus lymph node chronic lymphocytic leukemia cells correlates with survival capacity. Blood.

[67] Petlickovski A, Laurenti L, Li X, Marietti S, Chiusolo P, Sica S, Leone G, Efremov DG (2005). Sustained signaling through the B-cell receptor induces Mcl-1 and promotes survival of chronic lymphocytic leukemia B cells. Blood.

[68] Lopez-Matas M, Rodriguez-Justo M, Morilla R, Catovsky D, Matutes E (2000). Quantitative expression of CD23 and its ligand CD21 in chronic lymphocytic leukemia. Haematologica.

[69] Yaktapour N, Ubelhart R, Schuler J, Aumann K, Dierks C, Burger M, Pfeifer D, Jumaa H, Veelken H, Brummer T, Zirlik K (2013). Insulin-like growth factor-1 receptor (IGF1R) as a novel target in chronic lymphocytic leukemia. Blood.

[70] Li W, Saud SM, Young MR, Chen G, Hua B (2015). Targeting AMPK for cancer prevention and treatment. Oncotarget.

[71] Igata M, Motoshima H, Tsuruzoe K, Kojima K, Matsumura T, Kondo T, Taguchi T, Nakamaru K, Yano M, Kukidome D, Matsumoto K, Toyonaga T, Asano T, Nishikawa T, Araki E (2005). Adenosine monophosphate-activated protein kinase suppresses vascular smooth muscle cell proliferation through the inhibition of cell cycle progression. Circ Res.

[72] Pradelli LA, Beneteau M, Chauvin C, Jacquin MA, Marchetti S, Munoz-Pinedo C, Auberger P, Pende M, Ricci JE (2010). Glycolysis inhibition sensitizes tumor cells to death receptors-induced apoptosis by AMP kinase activation leading to Mcl-1 block in translation. Oncogene.

[73] Zhao Y, Altman BJ, Coloff JL, Herman CE, Jacobs SR, Wieman HL, Wofford JA, Dimascio LN, Ilkayeva O, Kelekar A, Reya T, Rathmell JC (2007). Glycogen synthase kinase 3alpha and 3beta mediate a glucose-sensitive antiapoptotic signaling pathway to stabilize Mcl-1. Mol Cell Biol.

[74] Aloj L, Caraco C, Jagoda E, Eckelman WC, Neumann RD (1999). Glut-1 and hexokinase expression: relationship with 2-fluoro-2-deoxy-D-glucose uptake in A431 and T47D cells in culture. Cancer Res.

[75] Salani B, Marini C, Rio AD, Ravera S, Massollo M, Orengo AM, Amaro A, Passalacqua M, Maffioli S, Pfeffer U, Cordera R, Maggi D, Sambuceti G (2013). Metformin impairs glucose consumption and survival in Calu-1 cells by direct inhibition of hexokinase-II. Sci Rep.

[76] Marini C, Salani B, Massollo M, Amaro A, Esposito AI, Orengo AM, Capitanio S, Emionite L, Riondato M, Bottoni G, Massara C, Boccardo S, Fabbi M, Campi C, Ravera S, Angelini G (2013). Direct inhibition of hexokinase activity by metformin at least partially impairs glucose metabolism and tumor growth in experimental breast cancer. Cell Cycle.

[77] Carew JS, Nawrocki ST, Xu RH, Dunner K, McConkey DJ, Wierda WG, Keating MJ, Huang P (2004). Increased mitochondrial biogenesis in primary leukemia cells: the role of endogenous nitric oxide and impact on sensitivity to fludarabine. Leukemia.

[78] van Delft MF, Wei AH, Mason KD, Vandenberg CJ, Chen L, Czabotar PE, Willis SN, Scott CL, Day CL, Cory S, Adams JM, Roberts AW, Huang DC (2006). The BH3 mimetic ABT-737 targets selective Bcl-2 proteins and efficiently induces apoptosis via Bak/Bax if Mcl-1 is neutralized. Cancer Cell.

[79] Del Gaizo Moore V, Brown JR, Certo M, Love TM, Novina CD, Letai A (2007). Chronic lymphocytic leukemia requires BCL2 to sequester prodeath BIM, explaining sensitivity to BCL2 antagonist ABT-737. J Clin Invest.

[80] Sum CF, Webster JM, Johnson AB, Catalano C, Cooper BG, Taylor R (1992). The effect of intravenous metformin on glucose metabolism during hyperglycaemia in type 2 diabetes. Diabet Med.

[81] Christensen MM, Brasch-Andersen C, Green H, Nielsen F, Damkier P, Beck-Nielsen H, Brosen K (2011). The pharmacogenetics of metformin and its impact on plasma metformin steady-state levels and glycosylated hemoglobin A1c. Pharmacogenet Genomics.

[82] Menendez JA, Oliveras-Ferraros C, Cufi S, Corominas-Faja B, Joven J, Martin-Castillo B, Vazquez-Martin A (2012). Metformin is synthetically lethal with glucose withdrawal in cancer cells. Cell Cycle.

[83] Ben Sahra I, Laurent K, Giuliano S, Larbret F, Ponzio G, Gounon P, Le Marchand-Brustel Y, Giorgetti-Peraldi S, Cormont M, Bertolotto C, Deckert M, Auberger P, Tanti JF, Bost F (2010). Targeting cancer cell metabolism: the combination of metformin and 2-deoxyglucose induces p53-dependent apoptosis in prostate cancer cells. Cancer Res.

[84] Cheong JH, Park ES, Liang J, Dennison JB, Tsavachidou D, Nguyen-Charles C, Wa Cheng K, Hall H, Zhang D, Lu Y, Ravoori M, Kundra V, Ajani J, Lee JS, Ki Hong W, Mills GB (2011). Dual inhibition of tumor energy pathway by 2-deoxyglucose and metformin is effective against a broad spectrum of preclinical cancer models. Mol Cancer Ther.

[85] Scotland S, Saland E, Skuli N, de Toni F, Boutzen H, Micklow E, Senegas I, Peyraud R, Peyriga L, Theodoro F, Dumon E, Martineau Y, Danet-Desnoyers G, Bono F, Rocher C, Levade T (2013). Mitochondrial energetic and AKT status mediate metabolic effects and apoptosis of metformin in human leukemic cells. Leukemia.

[86] Wilcock C, Bailey CJ (1994). Accumulation of metformin by tissues of the normal and diabetic mouse. Xenobiotica.

[87] Gong L, Goswami S, Giacomini KM, Altman RB, Klein TE (2012). Metformin pathways: pharmacokinetics and pharmacodynamics. Pharmacogenet Genomics.

[88] Cufi S, Corominas-Faja B, Lopez-Bonet E, Bonavia R, Pernas S, Lopez IA, Dorca J, Martinez S, Lopez NB, Fernandez SD, Cuyas E, Visa J, Rodriguez-Gallego E, Quirantes-Pine R, Segura-Carretero A, Joven J (2013). Dietary restriction-resistant human tumors harboring the PIK3CA-activating mutation H1047R are sensitive to metformin. Oncotarget.

[89] Blankart CR, Koch T, Linder R, Verheyen F, Schreyogg J, Stargardt T (2013). Cost of illness and economic burden of chronic lymphocytic leukemia. Orphanet J Rare Dis.

[90] Khan AE, Gallo V, Linseisen J, Kaaks R, Rohrmann S, Raaschou-Nielsen O, Tjonneland A, Johnsen HE, Overvad K, Bergmann MM, Boeing H, Benetou V, Psaltopoulou T, Trichopoulou A, Masala G, Mattiello A (2008). Diabetes and the risk of non-Hodgkin's lymphoma and multiple myeloma in the European Prospective Investigation into Cancer and Nutrition. Haematologica.

[91] Ghiotto F, Fais F, Tenca C, Tomati V, Morabito F, Casciaro S, Mumot A, Zoppoli G, Ciccone E, Parodi S, Bruno S (2009). Apoptosis of B-cell chronic lymphocytic leukemia cells induced by a novel BH3 peptidomimetic. Cancer Biol Ther.

[92] Chou TC, Talalay P (1984). Quantitative analysis of dose-effect relationships: the combined effects of multiple drugs or enzyme inhibitors. Adv Enzyme Regul.

