# Supplementary material for: Metformin inhibits cell cycle progression of B-cell chronic lymphocytic leukemia cells
Source: Oncotarget. 2015 Jun 5;6(26):22624–40. doi: 10.18632/oncotarget.4168 (PMC4673187; doi:10.18632/oncotarget.4168)
Supplement: Supplementary file 1 [file oncotarget-06-22624-s001.pdf]

SUPPLEMENTARY TABLES

Supplementary Table S1: CLL patient characteristics

Sample	Isotype	MUTATION	Rai stage	Treatment *	TTFT (months)	Alive/ Dead **	OS (months)	% of VH mutations	% of VL mutations	CD38 pos-neg
G025	μ	M-CLL	3	1	48	1	161,6	8,3	5	1
G062	μ	M-CLL	_	0	_	1	170	2,1	0,7	0
G085	μ	M-CLL	0	1	10	1	37	9	5	0
G087	γ	M-CLL	0	1	12	1	54	7,6	5,4	1
G110	μ	M-CLL	0	1	60	0	80	6,7	2,4	1
G117	μ	U-CLL	0	0	_	0	52			1
				* 1 = treated; 0 = untreated		** 1 = dead; 0 = alive				

Sample	VH gene	D gene	D gene rf	JH gene	VL gene	JL gene
G025	3–7	5–12		5	K3–15	1
G062	3–23	3–22		5	K3–15	1
G085	3–7*01	2–2*01	2	3*02	KV1–6*01	IGKJ3*01
G087	3–23*01	2–15*01	2	4*02	KV1–39*01	IGKJ4*01
G110	4–34*01	3–16*01	1	4*02	LV1–40*01	IGLJ1*01
